# Biocontrol Activity of *Bacillus altitudinis* CH05 and *Bacillus tropicus* CH13 Isolated from *Capsicum annuum* L. Seeds against Fungal Strains

**DOI:** 10.3390/microorganisms12101943

**Published:** 2024-09-25

**Authors:** Merle Ariadna Espinosa Bernal, Mayra Paola Mena Navarro, Jackeline Lizzeta Arvizu Gómez, Carlos Saldaña, Miguel Ángel Ramos López, Aldo Amaro Reyes, Monserrat Escamilla García, Juan Ramiro Pacheco Aguilar, Victor Pérez Moreno, José Alberto Rodríguez Morales, Erika Álvarez Hidalgo, Jorge Nuñez Ramírez, José Luis Hernández Flores, Juan Campos Guillén

**Affiliations:** 1 Facultad de Química, Universidad Autónoma de Querétaro, Cerro de las Campanas S/N, Querétaro 76010, Querétaro, Mexico; mespinosa06@alumnos.uaq.mx (M.A.E.B.); mmena27@alumnos.uaq.mx (M.P.M.N.); miguel.angel.ramos@uaq.mx (M.Á.R.L.); aldo.amaro@uaq.edu.mx (A.A.R.); monserrat.escamilla@uaq.edu.mx (M.E.G.); ramiro.pacheco@uaq.mx (J.R.P.A.); vperez@uaq.mx (V.P.M.); erika.beatriz.alvarez@uaq.mx (E.Á.H.); jorge.nunez@uaq.mx (J.N.R.); 2 Secretaría de Investigación y Posgrado, Centro Nayarita de Innovación y Transferencia de Tecnología (CENITT), Universidad Autónoma de Nayarit, Tepic 63173, Nayarit, Mexico; jackeline.arvizu@uan.edu.mx; 3 Facultad de Ciencias Naturales, Universidad Autónoma de Querétaro, Av. De las Ciencias S/N, Querétaro 76220, Querétaro, Mexico; carlos.saldana@uaq.mx; 4 Facultad de Ingeniería, Universidad Autónoma de Querétaro, Cerro de las Campanas S/N, Querétaro 76010, Querétaro, Mexico; jose.alberto.rodriguez@uaq.mx; 5 Centro de Investigación y de Estudios Avanzados del IPN, Irapuato 36824, Guanajuato, Mexico

**Keywords:** *Bacillus altitudinis*, *Bacillus tropicus*, volatile organic compound, biologic control, *Capsicum*, phytopathogenic fungi, genome sequencing

## Abstract

In this study, seed-surface-associated bacteria from fresh fruits of *Capsicum* spp. were analyzed to explore potential isolates for biocontrol of phytopathogenic fungal strains. A total of 76 bacterial isolates were obtained from three different species of chili pepper (*C. annuum* L., *C. pubescens* R. & P., and *C. chinense* Jacq.), and two isolates were selected via mycelial growth inhibition assays based on their production of volatile organic compounds (VOCs) against six fungal strains. Genomic analysis identified these isolates as *Bacillus altitudinis* CH05, with a chromosome size of 3,687,823 bp and with 41.25% G+C, and *Bacillus tropicus* CH13, with a chromosome size of 5,283,706 bp and with 35.24% G+C. Both bacterial strains showed high mycelial growth inhibition capacities against *Sclerotium rolfsii*, *Sclerotinia* sp., *Rhizoctonia solani*, and *Alternaria alternata* but lower inhibition capacities against *Colletotrichum gloesporoides* and *Fusarium oxysporum*. VOC identification was carried out after 24 h of fermentation with 64 VOCs for *B. altitudinis* CH05 and 53 VOCs for *B. tropicus* CH13. 2,5-Dimethyl pyrazine and acetoin had the highest relative abundance values in both bacterial strains. Our findings revealed that seed-surface-associated bacteria on *Capsicum* spp. have the metabolic ability to produce VOCs for biocontrol of fungal strains and have the potential to be used in sustainable agriculture.

## 1. Introduction

Chili pepper (*Capsicum* spp.) has been among the most important domesticated and cultivated plants in Mexico for approximately 6000 years [1,2,3]. Diverse genetic and phenotypic traits with commercial significance have been selected throughout the years, and today the diversity of the genus *Capsicum* is recognized with more than 30 species. Some of them are considered more economically relevant and are consequently cultivated extensively, such as *C. annuum* L. and other significant species [1,2,3,4,5,6]. Furthermore, these important species represent global production close to 34.5 million tons (Mt) with a market size that was valued at USD 4471.04 million in 2022 [7]. Production of fresh chili is led by China (16.7 Mt), México (2.8 Mt), Indonesia (2.7 Mt), Turkey (2.6 Mt), and Spain (1.5 Mt), while India is the largest producer of dry chili at 1.7 Mt [8].

However, these large economic gains are constantly affected by many plant diseases, causing significant reductions in plant growth, chili fruit yields, and seed production [9]. These diseases are generated under specific environmental conditions and are principally caused by fungi, bacteria, viruses, and nematodes, which cause severe losses in chili-producing countries [10,11,12,13]. Regarding fungal diseases, fruit rot caused by species of *Colletotrichum* has been reported in tropical and subtropical countries [14]. *Cercospora* leaf spot, or velvet spot, caused by *C. capsici* and *C. unonidicola* has been reported in countries with warm and moist conditions that promote these symptoms [15]. In the case of *Phytophthora capsici*, it affects roots and the lower portions of stems, leading to wilting. It is widespread in temperate and tropical countries as well as those with high humidity and summer rainfall [16]. Also, deaths of seedlings and subsequent reductions in plant stands (damping off) have been reported in chili-producing countries, which are principally caused by *Pythium* spp., *Fusarium* spp., and *Sclerotinia* spp. [17,18]. Another devastating disease in chili is the powdery mildew promoted by *Leveillula taurica* in countries with warm and dry climates [19]. On the other hand, *Rhizoctonia solani* is a major soil-borne pathogen of chili in chili-producing countries and causes root rot [11]. These fungal diseases are responsible for serious losses of up to 40% of chili production. For example, *F. oxysporum* produced up to 79% of deterioration in chili plants in Thailand and up to 56% in chili seedlings in Egypt [10]. In India, Korea, and Vietnam, it has been reported that between 10 and 80% of anthracnose disease is caused by *Colletotrichum* species during chili cultivation [11], while *P. capsici* has caused up to 100% yield losses during chili cultivation in tropical countries [16,20].

Nowadays, some single or combinatory strategies, such as cultural practices, chemical control, resistant varieties, and biological control, have been established in order to reduce the damage caused by pathogenic fungi in chili plant cultivars [9,10,11]. Although chemical control continues to be the most prevalent strategy used worldwide to ensure the global food supply, there are some restrictions on the use of some fungicides because they have been declared environmentally hazardous chemicals by the Environmental Protection Agency (EPA) and have created the problem of fungal resistance [21]. Thus, the excessive use of synthetic fungicides has had clear, direct impacts on soil, water, and non-target organisms [22]. In this scenario, the development and application of an alternative biological control method that uses beneficial microorganisms is necessary to reduce the environmental impact of chemical fungicides [23]. In this sense, research regarding the bacteria of the *Bacillus* genus has been relevant and interesting because this genus has multiple agroecological abilities. Diverse research works have shown that some members of the *Bacillus* genus, in particular *B. amyloquefaciens*, *B. pumilus*, *B. siamenses*, *B. subtilis*, *B. velezensis*, *B. safensis*, and *B. altitudinis* are capable of promoting plant growth, producing enzymes that add value within the industry (for example, xylanases and pectinases), inducing systematic resistance in plants, and synthesizing antimicrobial metabolites that provide plants protection and defense against phytopathogenic bacteria and fungi [24,25,26,27]. 

Seed-associated microorganisms with the metabolic ability to impact plant fitness or control pathogenic microorganisms have been studied. Nevertheless, whether secondary metabolites produced by chili fruits with antimicrobial effects during seed colonization by microorganisms can influence selection is unknown. In fact, capsaicin (8-methyl-N-vanillyl-6-nonenamide) is a significant component in the placenta of chili fruits, and some research works have demonstrated the antimicrobial effects of this secondary metabolite through inhibition of complex I (NADH dehydrogenase) during production of energy via the electron transport chain (ETC) [28,29]. However, alternative NADH dehydrogenase enzymes and degradative enzymes that reduce the capsaicin concentration have been reported in bacterial and fungal strains as mechanism to overcome this chili plant defense [30,31,32,33,34,35,36]. Therefore, studying bacterial colonization in chili pepper seeds with capsaicin resistance is important for evaluating their metabolic potential on plant fitness or as an alternative biocontrol method for phytopathogenic microorganisms with agricultural applications [37,38].

In this study, seed-surface-associated bacteria of *Capsicum* spp. fruits obtained from different regions of Mexico were isolated and characterized in order to evaluate interesting metabolic abilities as a potential alternative biocontrol method against fungal strains that cause diseases in chili cultivars. 

## 2. Materials and Methods

### 2.1. Isolation of Capsicum spp. Seed-Surface-Associated Bacteria

Fresh chili fruits of *Capsicum annuum* L., *Capsicum chinense* Jacq., and *Capsicum pubescens* R. & P. without evident signs of infection or visual damage were harvested from chili plants cultivated at Querétaro, Michoacán, and Yucatán, México. Afterwards, chili fruits were transported to the laboratory in sterile containers to carried out a surface-disinfection with 10% *v*/*v* of sodium hypochlorite during 10 min, followed with 70% *v*/*v* of ethanol for an additional 10 min. The fruits were air-dried under sterile conditions at room temperature. Once disinfected, the seeds were extracted from chili fruits and placed on a trypticasein soy agar (TSA) medium (Difco Laboratories, Detroit, MI, USA) and incubated at 37° for 24 h. Finally, grown colonies with different phenotypes were isolated in the same medium described above. 

### 2.2. Determination of the Antifungal Activity of Seed-Surface-Associated Bacteria In Vitro

The fungal strains: *Sclerotinia* sp., *Rhizoctonia solani*, *Sclerotium rolfsii*, *Alternaria alternata*, *Fusarium oxysporum*, and *Colletotrichum gloeosporioides* were obtained from the Laboratory of Plants and Agricultural Biotechnology of the Autonomous University of Querétaro, Mexico. Inhibition assays of radial mycelial growth of these fungal strains were evaluated through the production of volatile organic compounds (VOCs) of 76 seed-surface-associated bacterial isolates obtained according to the methodology previously described [37]. In general, each fungal disc (with diameter of 7 mm) was inoculated in a Petri dish of 9 cm of diameter with potato dextrose agar (PDA) medium (Difco Laboratories, Detroit, MI, USA), while that in other similar Petri dish with TSA medium (Difco Laboratories, Detroit, MI, USA) was inoculated each seed-surface-associated bacteria isolate with a bacterial suspension of 1 × 10^8^ CFU/mL. Afterwards, both Petri dishes cultures, where the fungal strain was in the upper side and the bacterial culture was in the lower side, were merged and sealed with parafilm and incubated for a period of 5–7 days at 28 °C in accordance with each fungal growth rate. For the control, only the fungal disc was inoculated in the upper side without the bacterial strain in the lower side. During the incubation period, measurements of radial mycelial growth were recorded using the equation ICM = [(C) − (T)/C] × 100%. The letters C and T refer to the diameters (cm) of radial mycelial in the control and treatments. 

### 2.3. Genome Sequencing, Assembly, and Annotation

Based on the inhibition assays against the fungal strains, we selected the bacterial strains CH05 and CH13 since they presented the highest inhibition rates, and we decided to identify them via genome sequencing. Both bacterial strains were grown in a TSA medium (Difco Laboratories, Detroit, MI, USA) for 24 h at 37 °C and harvested for DNA purification using a ZymoBIOMICS^TM^ DNA Miniprep Kit (Zymo Research, Irvine, CA, USA). Then, a NovaSeq^®^ (Illumina, San Diego, CA, USA) platform at Zymo Research, Irvine, CA, USA, was used for genome sequencing. The sequence reads obtained were analyzed at the public-free platform Bacterial and Viral Bioinformatics Resource Center (BV-BRC) [39]. The quality of the sequences was obtained via a filtering and trimming step using the Fastq Utilities Service [39]. Then, using the comprehensive genome analysis service at PATRIC [39], the genomes of the isolates CH05 and CH13 were assembled with SPAdes [40] and annotated using the RAST tool RASTtk—v1.073 [41]. The genome map was obtained with Proksee Assemble, CGView Builder, CARD RGI, and Prokka [42]. The Codon Tree analysis service at BV-BRC was used to construct a bacterial phylogenetic tree. This method utilized Mash/MinHash [43], PATRIC global protein families (PGFams) [39], MUSCLE v5 [44], and RaxML v8.2.11 (Randomized Axelerated Maximum Likelihood) [45]. The Average Nucleotide Identities (ANIs) was analyzed with JSpeciesWS [46] to calculate the probability that the bacterial genomes belonged to the similar species via a pairwise comparison.

DNA sequences were deposited in the NCBI as BioProject PRJNA1143437 with accession number JBGCTZ000000000 for *B. altitudinis* CH05. For *B. tropicus* CH13, sequences were deposited as BioProject PRJNA1143476 with accession number JBGCUA000000000. 

### 2.4. Antibiotic Sensitivity

Antimicrobial resistance and spread of these molecular mechanisms could be a negative trait in bacterial strains with potential metabolic abilities to be used in agricultural systems. For this reason, we evaluated antibiotic resistance to gain insight into better decision. Antibiotic sensitivity was evaluated using the disk diffusion method based on the CLSI (Clinical and Laboratory Standards Institute: CLSI Guidelines) guide [47]. A bacterial suspension of *B. altitudinis* CH05 and *B. tropicus* CH13 was inoculated on the surface of a Mueller–Hinton agar medium (BD Bioxon, Ciudad de Mexico, Mexico). Finally, the following antimicrobials were tested: ampicillin (10 µg), carbenicillin (100 µg), penicillin (10 U), clindamycin (30 µg), dicloxacillin (1 µg), erythromycin (15 µg), tetracycline (30 µg), vancomycin (30 µg), amikacin (30 µg), cephalothin (30 µg), cefotaxime (30 µg), ciprofloxacin (5 µg), chloramphenicol (30 µg), gentamicin (10 µg), netilmicin (30 µg), nitrofurantoin (300 µg), norfloxacin (10 µg), and sulfamethoxazole/trimethoprim (25 µg). The bacterial culture plates were incubated at 37 °C for 24 h, and measurements of the complete inhibition of antibiotics, represented by clear zones were register in millimeters by duplicate.

### 2.5. Analysis of VOCs Produced by B. altitudinis CH05 and B. tropicus CH13 via HS-SPME-GC-MS

In order to determine if VOCs were produced by the *B. altitudinis* CH05 and *B. tropicus* CH13 strains, both strains were grown in a tryptone soy broth (TSB) medium at 37 °C and 120 rpm during 48 h. From these cultures, 4 mL were obtained every 12 h for 48 h. The procedure to obtain cell-free filtrates was carried out via centrifugation at 14,000 rpm for 5 min (performed twice) and filtered through a sterile membrane of 0.22 µm (Sigma Aldrich, St. Louis, MO, USA). Afterwards, to evaluate the VOCs produced during this period, the filtrates obtained at 12, 24, 36, and 48 h were evaluated as follows: First, 500 μL of a filtrate was plated on the PDA medium (Difco Laboratories, Detroit, MI, USA) and inoculated with a mycelial disk (7 mm) of the fungus *Screrotium rolfsii*. Then, inhibition of radial growth was measured according to the equation described above. The samples extracted at 24 h from both bacterial strains demonstrated major inhibition of growth of the fungus and, therefore, were selected for the characterization of VOCs following the methodology reported previously [37]. 

### 2.6. Evaluation of Standard VOCs against Phytopathogenic Fungi

The antifungal activity of four commercially available VOCs (2,5 dimethylpyrazine (98%, Sigma-Aldrich, USA), benzaldehyde (97%, Sigma-Aldrich, USA), acetoin (≥95%, Sigma-Aldrich, USA), and 2-butanone (≥99%, Sigma-Aldrich, USA)) was determined. These standard VOCs were tested in order to evaluate their antagonistic effects against the growth of two fungal strains (*S. rolfsii* and *R. solani*). First, a Petri dish of 9 cm of diameter was used to place a sterile filter paper discs containing the synthetic VOCs, while in other Petri dish with potato dextrose agar (PDA) medium (Difco Laboratories, Detroit, MI, USA) was inoculated each fungal disc (with diameter of 7 mm). Afterwards, both Petri dishes with the fungal strain in the upper side and synthetic VOCs in the lower side, were merged and sealed with parafilm and incubated for a period of 5–7 days at 28 °C. The treatments tested included T1, T2, and T3, which contained 100, 150, and 200 µL of 2,5-dimethyl-pyrazine, respectively. The treatments with acetoin were T4 (100 µL), T5 (150 µL), and T6 (200 µL). The treatments with benzaldehyde were T7 (10 µL) and T8 (20 µL). The treatments with 2-butanone were T9 (100 µL), T10 (150 µL), and T11 (200 µL). Then, combinations of pyrazine, acetoin, and benzaldehyde were prepared with different volume concentrations. Treatment T12 contained 10 µL of each VOC; treatment T13 contained 15 µL of each VOC; and treatment T14 contained 10 µL of 2,5 dimethylpyrazine, 20 µL of acetoin, and 20 µL of benzaldehyde. In the control plates, only the fungal discs were used without the standard VOCs.

### 2.7. Statistical Analysis

The experiments of radial mycelial growth inhibition with VOCs production from bacterial isolates were carried out at least three biological replicates for statistical analysis, and the data were analyzed using Minitab version 18.0. Means with ± standard error were analyzed via one-way ANOVA (*p* < 0.05).

## 3. Results

### 3.1. Isolation and Characterization of Seed-Surface-Associated Bacteria

**Figure 1:**
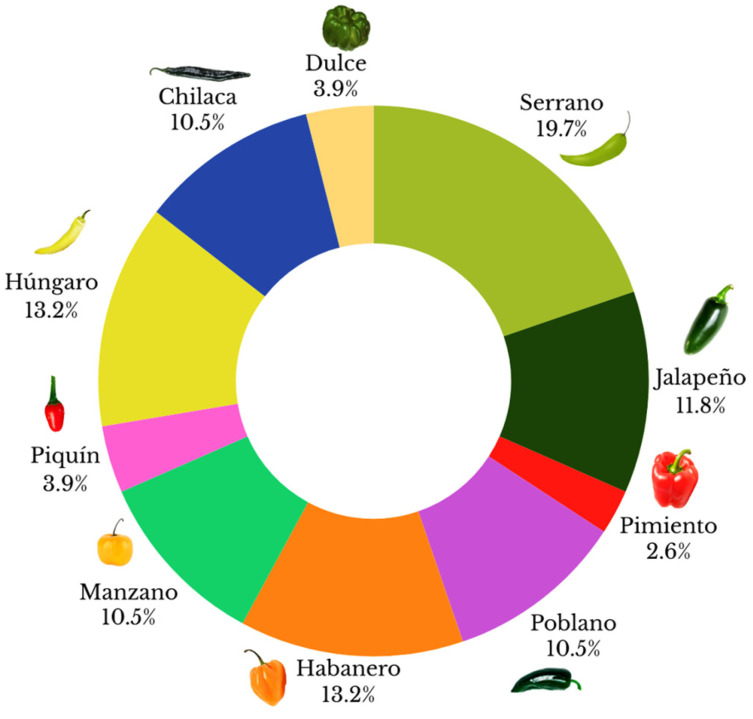
Distribution of seed-surface-associated bacteria obtained from varieties of chili: *C. annuum* L. (Serrano, Jalapeño, Pimiento, Poblano, Piquin, húngaro, Chilaca, and Dulce), *C. pubescens* (manzano), and *C. chinense* (habanero).

**Figure 2:**
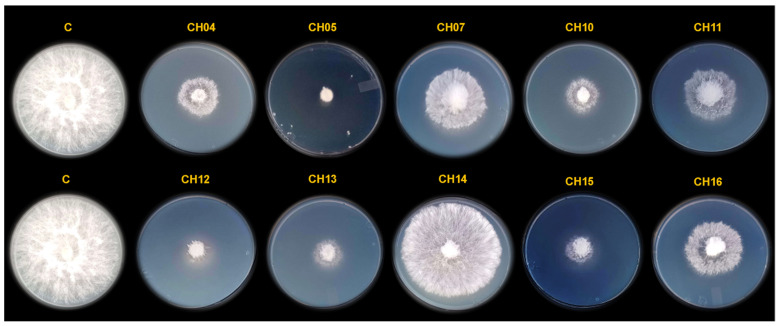
Inhibition of mycelial growth of *S. rolfsii* by VOCs produced by bacterial isolates. From 76 bacterial isolates, we only show results from 10 bacterial isolates selected, and the untreated controls are indicated by C.

Initially, a total of 76 bacterial isolates were obtained from seeds of three different chili pepper species (Table 1): *C. annuum* L. (58 isolates from the varieties serrano, jalapeño, pimiento, poblano, piquin, húngaro, chilaca, and dulce), *C. pubescens* (8 isolates from the variety manzano), and *C. chinense* (10 isolates from the variety habanero). The distribution of these isolates is shown in Figure 1. In addition, antifungal activity against *S. rofsii* was assessed through VOC production as a first step to select bacterial isolates with inhibitory ability. Of these 76 isolates, only 10 obtained from serrano pepper seeds (*C. annuum* L.) inhibited the radial mycelial growth of *S. rolfsii* under these assays’ conditions. Figure 2 shows the inhibition of radial mycelial growth by these 10 bacterial isolates selected, the mean value of inhibition rates was 23.75% (CH14), 50% (CH07), 51.25% (CH11), 53.75% (CH16), 66.25% (CH04), 67.5% (CH10), 68.75% (CH13), 73.75% (CH15), 76.25% (CH12), and 77.5% (CH05), in that order. 

### 3.2. Characterization of Bacterial Isolates CH05 and CH13 against Phytopathogenic Fungal Strains In Vitro

**Figure 3:**
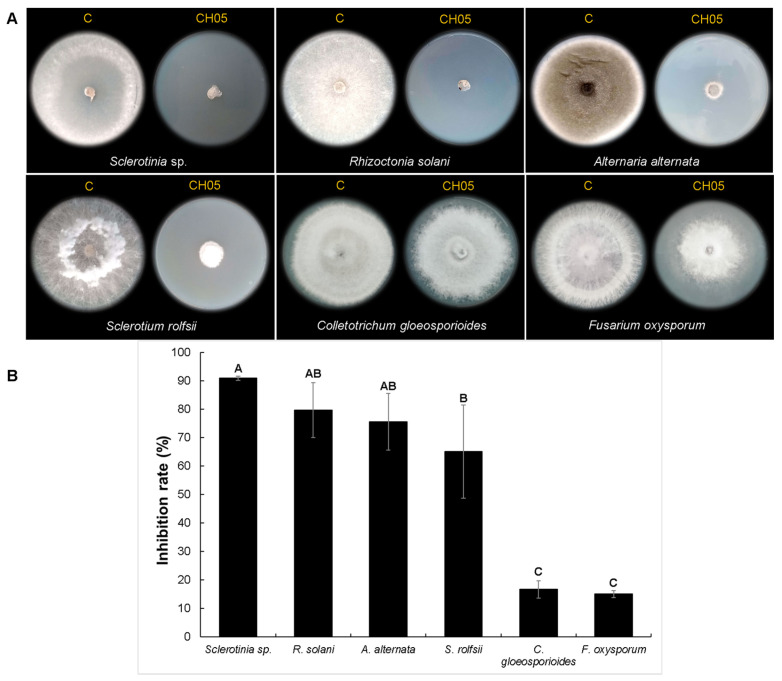
In vitro evaluation of mycelial growth inhibition by the bacterial CH05 isolate against fungal strains. (**A**) shows the radial mycelial growth of each fungal strain tested against the VOCs produced by the bacterial isolate. Untreated controls are indicated by the letter C. (**B**) shows the percentage of mycelial growth inhibition for each fungal strain. Statistical analyses were analyzed by one-way ANOVA and Tukey’s test. Statistical differences are represented by letters (*p* ˂ 0.05).

**Figure 4:**
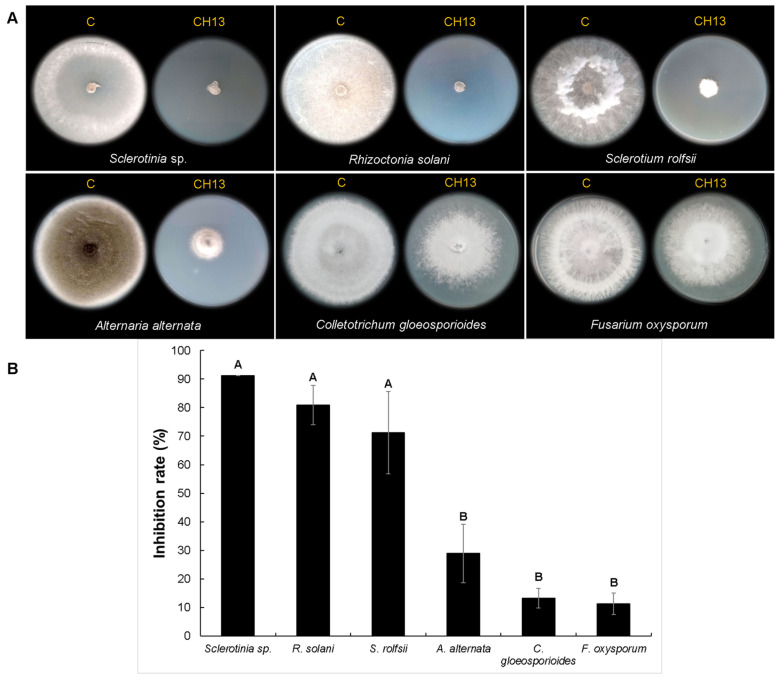
In vitro evaluation of mycelial growth inhibition by the bacterial CH13 isolate against fungal strains. (**A**) shows the radial mycelial growth of each fungal strain tested against the VOCs produced by the bacterial isolate. Untreated controls are indicated by the letter C. (**B**) shows the percentage of mycelial growth inhibition for each fungal strain. Statistical analyses were analyzed by one-way ANOVA and Tukey’s test. Statistical differences are represented by letters (*p* ˂ 0.05).

Based on its bacterial phenotype and because it had the highest fungal inhibition rate, we selected the bacterial isolate CH05 obtained from serrano pepper seeds at Querétaro region. CH13 was selected randomly from a group of bacterial isolates (CH10, CH12, CH13, and CH15) that showed similar colony phenotypes and was obtained from serrano pepper seeds at Michoacán. Both bacterial isolates were tested against additional fungal strains (*Sclerotinia* sp., *Rhizoctonia solani*, *Alternaria alternata*, *Colletotrichum gloeosporioides*, and *Fusarium oxysporum*) using double-culture assays (Figure 3 and Figure 4). The results in Figure 3 show that the colony diameters of all tested fungal strains were affected by the VOCs produced by the CH05 isolate during growth on a TSA medium compared with the untreated control. The highest mycelial growth inhibition percentage was achieved by *Sclerotinia* sp. at 90.83%, followed by *R. solani* at 79.58%, *A. alternata* at 75.46%, and *S. rolfsii* at 65%. Meanwhile, *F. oxysporum* and *C. gloeosporioides* presented low mycelial growth inhibition percentages of 18.52% and 16.62%, respectively. On the other hand, the production of VOCs by the bacterial isolate CH13 on a TSA medium (Figure 4) showed a similar high inhibition rate against *Sclerotinia* sp. of 91.25%, followed by *R. solani* at 80.83% and *S. rolfsii* at 71.25%. *A. alternata*, *C. gloeosporioides*, and *F. oxysporum* exhibited lower mycelial inhibition rates of 28.88%, 13.8%, and 10.37%, respectively. Based on these results, we decided to analyze both bacterial isolates using genome sequencing and VOC composition profiles.

### 3.3. Genomic Analysis of B. altitudinis CH05 and B. tropicus CH13

**Figure 5:**
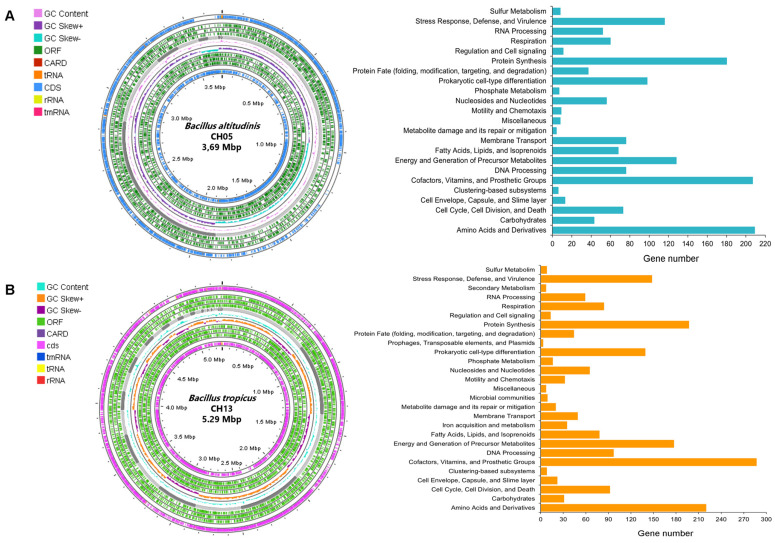
Circular genomic map and subsystem information for *B. altitudinis* CH05 (**A**) and *B. tropicus* CH13 (**B**). The rings, from outside to inside, indicate contigs, CDSs on the forward strand, CDSs on the reverse strand, RNA genes, CDSs similar to known antimicrobial resistance genes, CDSs similar to known virulence factors, the GC content, and the GC skew. A representation of the assignments of the functional subsystems is shown on the right side of the chromosome figure.

The bacterial isolates CH05 and CH03 were identified via genome analysis as *B. altitudinis* and *B. tropicus*, respectively. The bacterial genomes of the *B. altitudinis* CH05 and *B. tropicus* CH13 strains had estimated sizes of 3,687,823 bp and 5,283,706 bp and average guanine and cytosine contents of 41.25% and 35.24%, respectively (Table 2). The genome quality assessment of *B. altitudinis* CH05 showed coarse consistency (98.2), fine consistency (96.7), CheckM completeness (100), and CheckM contamination (0.1), which indicated the high-quality genome. *B. tropicus* CH13 showed coarse consistency (99.8), fine consistency (98.5), and CheckM completeness (100), which indicated a high-quality genome. In addition, the annotation results indicated that they had 3894 and 5508 protein coding sequences (CDSs), respectively. The genomes of *B. altitudinis* CH05 and *B. tropicus* CH13 were annotated using the RAST toolkit (RASTtk) and were found to contain specific genes (Table 2). These genomes are represented by genes related with virulence factors, antibiotic resistance genes, transporters and drug target genes, which were all identified in different databases. Annotation of the proteins indicated that they are specialized in various biological processes at the molecular and cellular levels. Furthermore, functional annotation of the genomes of *B. altitudinis* CH05 and *B. tropicus* CH13 based on the PATRIC source revealed the genes that encode various cellular processes, cell signaling, regulation, and metabolic pathways (Figure 5).

An analysis of the annotation of the genome of *B. tropicus* CH13, according to a database provided by bacterial virulence factors (VDFB), indicated that the genes related to virulence were classified within mechanisms of action such as toxins, pore-forming toxins, and enzyme metalloproteases. Genes that can be highlighted include *nheA*, *nheB*, and *nheC* (non-hemolytic enterotoxins); *hblA*, *hblC*, and *hblD* (hemolytic enterotoxins); cytotoxin K (*cytK*); and immune inhibitor A (*inhA*) (a metalloprotease). Within the antibiotic resistance genes, both genomes matched with the PATRIC database. For example, the *gyrA* and *gyrB* genes (ATP-dependent DNA gyrases) had mutations that confer resistance to the fluoroquinolone class of antibiotics. Also, the *murA* gene encodes an enzyme involved in the synthesis of peptidoglycans and, when mutated, promotes resistance to the antibiotic fosfomycin. Daptomycin resistance is associated with the *pgsA* gene. The *alr* gene encodes the enzyme D-alanine racemase. It is involved in the synthesis of peptidoglycan, and overexpression confers resistance to the antibiotic D cycloserine. Also, the *dxr* gene favors resistance to the antibiotic fosmidomycin. The *folA* and *dfr* genes both encode dihydrofolate reductase, an enzyme that promotes resistance to the antibiotic trimethoprim. The *folP* gene encodes the enzyme di-hydro-pteroate synthase (DHPS), and it is known that *folP* mutations lead to resistance to the sulfonamide class of antibiotics. According to the PATRIC source, *B. tropicus* CH13 contained the *Bcll* family, which produces a broad-spectrum subclass B1 beta-lactamase with the ability to hydrolyze a large number of antibiotics belonging to the classes of cephalosporins and penams. Additional antibiotic resistance-related genes in both genomes can be seen in the Supplementary Materials (Figures S1 and S2). With regard to these genotypes and as an approximation to determine some traits of these bacterial strains and future applications, the AMR phenotype was evaluated. It was found that *B. altitudinis* CH05 showed resistance to five of the eighteen antibiotics tested, including ampicillin, carbenicillin, cefotaxime, dicloxacillin, and penicillin. Interestingly, *B. tropicus* CH13 was sensitive to all antibiotics, which suggested that an AMR phenotype is necessary for better interpretation, as was the case for beta-lactamase detection in the genome, but beta-lactam resistance was not observed, probably due to an unfunctional gene. 

**Figure 6:**
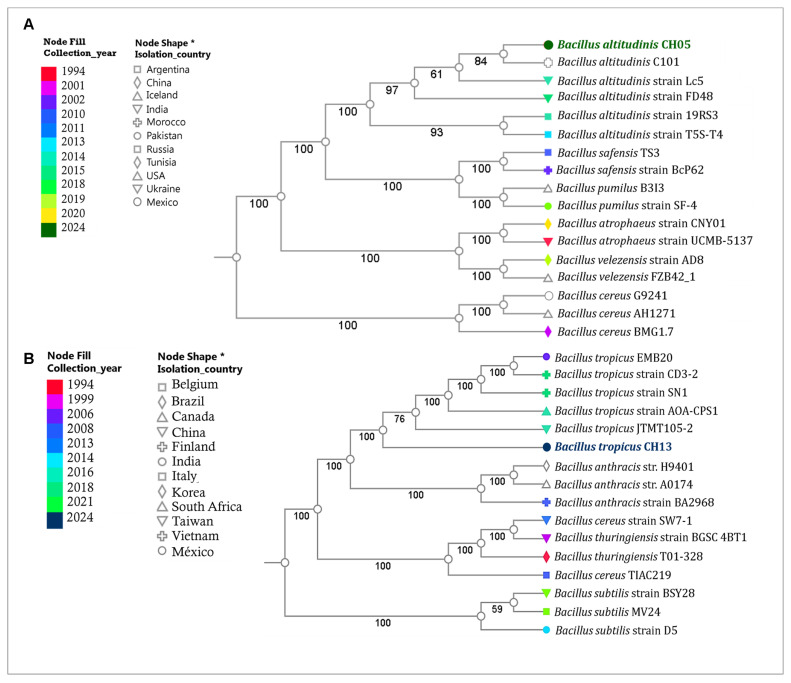
Phylogenetic trees of *B. altitudinis* CH05 (**A**) and *B. tropicus* CH13 (**B**) based on 100 core genes. In this figure, the years and places where the strains were isolated are indicated by colors and node shape *.

According to a phylogenetic analysis (Figure 6) that consisted of 100 core genes, we found that *B. altitudinis* CH05 matches related species (Figure 6A), including *B. altitudinis* strain C101, *B. altitudinis* strain Lc5, *B. altitudinis* strain FD48, *B. altitudinis* strain 19RS3, and *B. altitudinis* strain T5S-T4. In the case of *B. tropicus* CH13, it is a member of the *Bacillus cereus* group and is in a clade (Figure 6B) with *B. tropicus* strain EMB20, *B. tropicus* strain CD3-2, *B. tropicus* strain SN1, *B. tropicus* strain AOA-CPS1, and *B. tropicus* JTM105-2. The ANI values confirmed these phylogenetic results for *B. altitudinis* CH05 and *B. tropicus* CH13 (Tables S1 and S2 in the Supplementary Materials). A pairwise genome comparison of *B. altitudinis* CH05 showed ANI values of >97% with other related *B. altitudinis* strains. In the case of *B. tropicus* CH13 showed ANI values of 95.69% with the *B. tropicus* N24 strain, 95.71% with the *B. cereus* Q1 strain, 94.49% with the *B. anthracis* HYU01 strain, and 91.05% with the *B. thuringiensis* HD-789 strain. 

### 3.4. Characterization of VOCs

**Figure 7:**
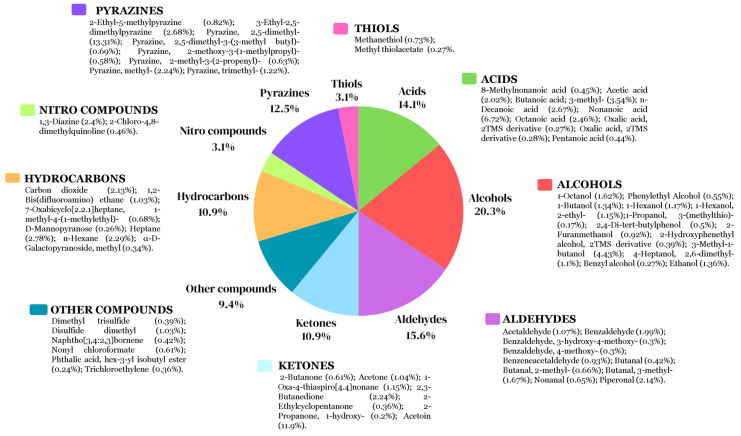
The percentages of different classes of VOCs produced by *B. altitudinis* CH05 after 24 h of fermentation. The relative area percentage of each VOC is shown in parentheses.

Based on the results of mycelial growth inhibition using cell-free filtrates obtained from both bacterial strains over 48 h, we decided to analyze the profiles of the VOCs produced after 24 h because they were the samples with the highest mean inhibition rates for the tested fungal strains (50% for *B. altitudinis* CH05 and 60% for *B. tropicus* CH13). The rest of the cell-free filtrates had mycelial growth inhibition rates below 20%. In the case of *B. altitudinis* CH05, a total of 64 compounds were identified (Figure 7 and Supplementary Table S3) and classified as alcohols (20.3%), aldehydes (15.6%), acids (14.1%), pyrazines (12.5%), ketones (10.9%), hydrocarbons (10.9%), nitro compounds (3.1%), thiols (3.1%), and other compounds (9.4%).

The compounds with highest relative abundance values (%) correspond to the following VOCs (Figure 7): 2,5-dimethyl pyrazine (13.31%), acetoin (11.19%), nonanoic acid (6.72%), 3-methyl-1-butanol (4.43%), butanoic acid, 3-methyl (3.54%), 3-ethyl-2, 5-dimethyl pyrazine (2.68%), n-decanoic acid (2.67%), n-decanoic acid (2.67%), octanoic acid (2.46%), 1,3-diazine (2.40%), methyl pyrazine (2.24%), 2,3-butanedione (2.24%), piperonal (2.14%), benzaldehyde (1.99%), 3-butanol methyl (1.67%), ethanol (1.36%), and 1-butanol (1.34%).

**Figure 8:**
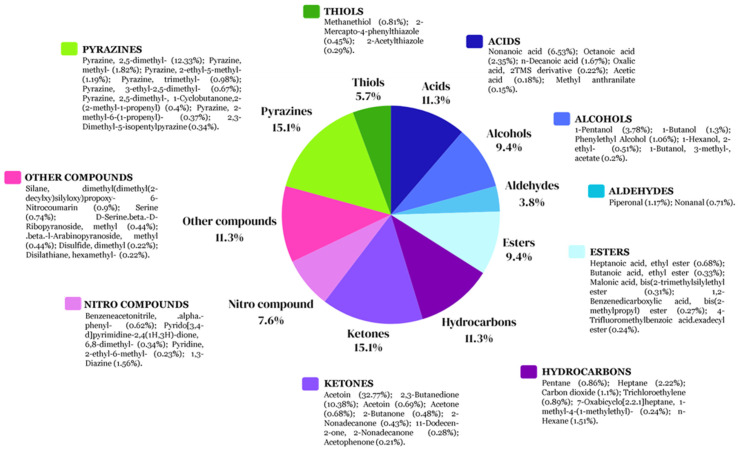
The percentages of different classes of VOCs produced by *B. tropicus* CH13 after 24 h of fermentation. The relative area percentage of each VOC is shown in parentheses.

On the other hand, 53 VOCs were identified in *B. tropicus* CH13 and were classified as pyrazines (15.1%), ketones (15.1%), acids (11.3%), hydrocarbons (11.3%), alcohols (9.4%), esters (9.4%), nitro compounds (7.6%), thiols (5.7%), aldehydes (3.8%), and other compounds (11.3%). According to the obtained data, the main compounds that presented the highest relative peak areas (%) were acetoin (32.77%), 2,5 dimethyl pyrazine (12.33%), 2,3-butanedione (10.38%), nonanoic acid (6.53%), 1-pentanol (3.78%), octanoic acid (2.35%), methyl pyrazine (1.82%), n-decanoic acid (1.67%), n-hexane (1.51%), 1-butanol (1.30%), 2-ethyl-5 methylpyrazine (1.19%), and piperonal (1.17%), as shown in Figure 8 and Supplementary Table S4.

### 3.5. Mycelial Growth Effects of Standard VOCs

**Figure 9:**
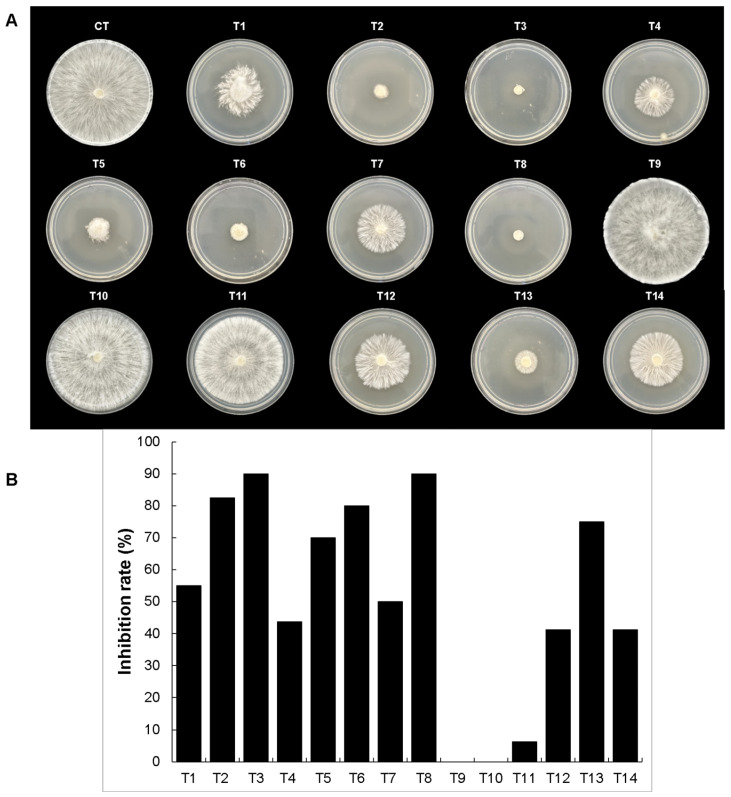
The mycelial growth inhibition rates of standard VOCs against *S. rolfsii*. The treatments applied were T1, T2, and T3 with 2,5 dimethyl pyrazine concentrations of 100 µL, 150 µL, and 200 µL; T4, T5, and T6 with 100 µL, 150 µL, and 200 µL of acetoin; T7 and T8 with 10 µL and 20 µL of benzaldehyde; and T9, T10, and T11 with 50 µL, 100 µL, and 150 µL of 2-butanone. Also, the combination of pyrazine, acetoin, and benzaldehyde was used with different concentrations. T12 used 10 µL of each compound, T13 used 15 µL of each compound, and T14 used 10 µL of 2,5 dimethyl pyrazine and 20 µL of the other compounds. (**A**) The radial mycelial growth inhibition effects of the VOCs used against *S. rolfsii*. (**B**) Percentages of mycelial inhibition with the mean value of two essays. CT mean is the control without treatment.

**Figure 10:**
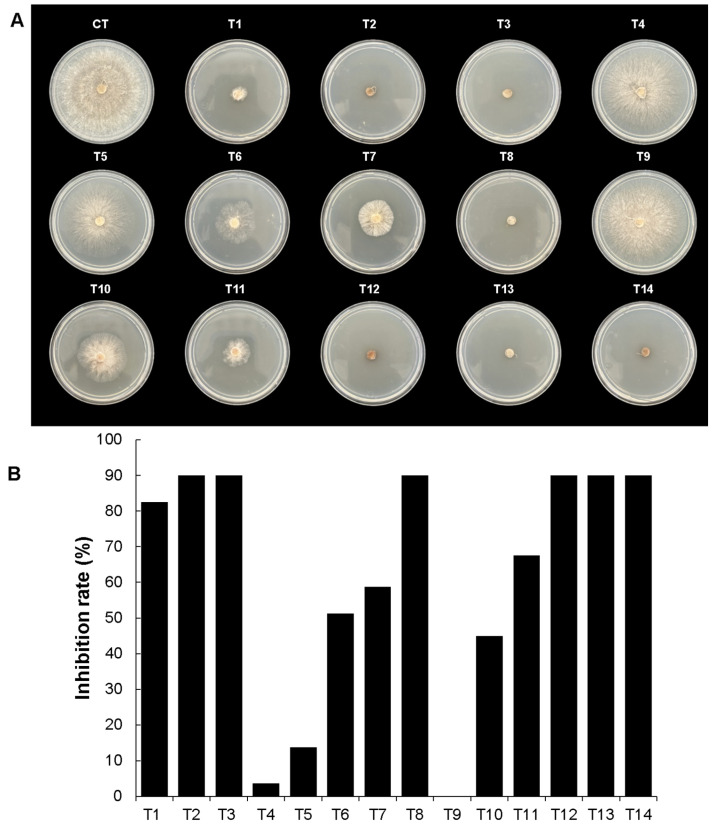
The mycelial growth inhibition rates of standard VOCs against *R. solani*. The treatments applied were T1, T2, and T3 with 2,5 dimethyl pyrazine concentrations of 100 µL, 150 µL, and 200 µL; T4, T5, and T6 with 100 µL, 150 µL, and 200 µL of acetoin; T7 and T8 with 10 µL and 20 µL of benzaldehyde; and T9, T10, and T11 with 50 µL, 100 µL, and 150 µL of 2-butanone. Also, the combination of pyrazine, acetoin, and benzaldehyde was used with different concentrations. T12 used 10 µL of each compound, T13 used 15 µL of each compound, and T14 used 10 µL of 2,5 dimethyl pyrazine and 20 µL of the other compounds. (**A**) The radial mycelial growth inhibition effects of the VOCs used against *R. solani*. (**B**). Percentages of mycelial inhibition with the mean value of two essays. CT mean is the control without treatment.

According to the VOC results, two compounds presented the highest relative peak areas in both bacterial strains: acetoin and 2,5 dimethyl pyrazine. To understand their individual effects on mycelial growth, both compounds were obtained commercially and tested on *S. rolfsii* and *R. solani* for comparison. As indicated in Figure 9, for *S. rolfsii*, 2,5 dimethyl pyrazine exhibited high mycelial growth inhibition rates of 82.5% and 90% at volume concentrations of 150 µL (T2) and 200 µL (T3), respectively, while a mycelial growth inhibition rate of only 53% was observed at a concentration of 100 μL (T1) when compared with the untreated control. On the other hand, acetoin presented a mycelial growth inhibition rate of 44% in the 100 µL treatment (T4), followed mycelial growth inhibition rates of 70% and 80% at concentrations of 150 µL (T5) and 200 μL (T6), respectively. Additionally, for comparison, we used two other commercially available compounds with antifungal properties that were detected in VOCs from *B. altitudinis* CH05 and *B. tropicus* CH13: 2-butanone and benzaldehyde. The results showed that benzaldehyde presented a mycelial growth inhibition rate of 50% in the 10 µL treatment (T7) and a rate of 90% in the 20 μL treatment (T8). 2-Butanone showed a mycelial growth inhibition rate of 6% at a concentration of 150 μL (T11). However, there was no inhibition in the 50 µL and 100 μL treatments (T9 and T10). Based on these results, 2,5 dimethyl pyrazine, acetoin, and benzaldehyde were mixed at different volumes to evaluate such formulations and their effects on mycelial growth inhibition. Treatment T12, which contained 10 μL of these compounds, showed a mycelial growth inhibition rate of 40%. Treatment T13, with 15 µL of these compounds, showed a mycelial growth inhibition rate of 75%. Interestingly, for treatment T14, where the 2,5 dimethyl pyrazine was reduced to 10 µL and the other compounds were increased to 20 µL, the mycelial growth inhibition rate was only 41%. Therefore, combinations of VOCs and concentrations demonstrated important mycelial growth reductions. Additionally, we tested *R. solani*. Figure 10 shows that 2,5 dimethyl pyrazine exhibited a high mycelial growth inhibition rate of 82.5% when treated with 100 µL (T1) and a rate of 90% at volume concentrations of 150 µL (T2) and 200 µL (T3). Acetoin had a lower capacity for mycelial growth inhibition. Thus, the treatment with 100 µL (T4) only had an inhibition rate of 4%, the treatment with 150 μL (T5) had a rate of 14%, and an average rate of 51% was observed with 200 µL (T6). In the case of benzaldehyde, it had a mycelial growth inhibition rate of 59% in the 10 μL treatment (T7) and a rate of 90% in the 20 μL (T8) treatment. On the other hand, 2-butanone presented a lower mycelial growth inhibition rate of 5% at 50 µL (T9) but major mycelial growth inhibition rates of 45% and 67.5% at 100 µL (T10) and 150 μL (T11), respectively. Interestingly, all volume concentrations of the combination of pyrazine, acetoin, and benzaldehyde presented a similar high rate of mycelial growth inhibition of 90% (T12, T13, and T14). These results show that each fungal strain presents different VOC effects and that a complex mix of these compounds can be used to control mycelial growth with better results. 

## 4. Discussion

In the present study, seed-surface-associated bacterial strains were isolated from *Capsicum* spp. fruits and characterized. From these isolates, two bacterial strains were selected because they had the highest mycelial growth inhibition activity against *Sclerotinia* sp. *R. solani*, *S. rolfsii*, and *A. alternata* through the production of VOCs. Genome analysis revealed that these strains belong to the *Bacillaceae* family, and they were matched with the closest species in the *Bacillus* genus. Based on a phylogenetic analysis with 100 core genes and ANI values, we confirmed their identities to be *B. altitudinis* CH05 and *B. tropicus* CH13, which represent important bacterial species reported from chili seeds. For both bacterial strains, genome mining indicated gene clusters related to metabolic pathways involved in the production of VOCs. Therefore, our findings provide evidence that chili seed-surface-associated bacteria possess mechanisms conserved among diverse species of the *Bacillus* genus that might control phytopathogenic fungal strains [48]. 

*B. altitudinis* is a member of the *B. pumilus* group and has relatives that belong to the *Subtilis* clade, which include relevant and well-studied species with applications in agriculture [49]. As an example of these applications, *B. altitudinis* is widely dispersed in the environment and has been isolated from grapevine leaves as an endophyte. It has the ability to control grapevine downy mildew caused by *Plassmopara viticola* [50]. In another study, the *B. altitudinis* JSCX-1 strain was isolated from soybean rhizosphere, and its characterization demonstrated mycelial growth inhibition and plant resistance against the plant pathogen *Phytophthora sojae* (Kaufmann and Gerdemann), which causes destructive disease in soybeans [51]. Also, the *B. altitudis* KRS010 strain was isolated from seeds of the *Gossypium hirsutum* cultivar “Zhongzhimian No. 2.”. This strain exhibited VOC production and the ability to reduce mycelial growth of various pathogenic fungi such as *Fusarium* spp., *Colletotrichum* spp., *Verticillium dahliae*, *Botrytis cinerea*, and *Magnaporthe oryzae* [52]. Production and identification of lipopeptides were reported for the *B. altitudinis* TM22A strain, which has the ability to reduce mycelial growth and conidial germination on *A. alternata* in vitro [53]. Another important endophytic bacterial strain was isolated from healthy tea leaves (*Camellia sinensis* (L.) O. Kuntze) and identified as *B. altitudinis* GS-16. It has the important metabolic ability to produce active molecules with antifungal properties against *F. equiseti* B-3-1, *F. graminearum* Z-16, *F. oxysporum* X1-16, *C. gloeosporioides* 1-F, *C. camelliae* Tj-26, *Plectosphaerella cucumerina* Z-14, *F. dimerum* X1-19 and *A. alternata* A-3 [54]. In addition, endophytic bacteria isolated from different tissues of Bhut Jolokia (*Capsicum chinense* Jacq.), one of the hottest chilies in the world, were identified as members of the *Bacillus* genus. One of them, identified as *B. altitudinis*, showed the strongest inhibition of mycelial growth of *C. gloeosporioides* [55]. Therefore, these findings demonstrate the potential of *B. altitudinis* in the biocontrol of phytopathogenic fungal strains and as important bacteria associated with plant tissue. Our results contribute to our understanding of chili seed-surface-associated *B. altitudinis* CH05, which has relevant metabolic traits. It produces VOCs that affect the mycelial growth of fungal strains and has the potential to be used in sustainable agriculture. 

On the other hand, *B. tropicus* is a novel species that was proposed recently and is a member of the *B. cereus* group [56]. Although diverse species from this bacterial group cause illnesses in humans and animals due to the production of toxins [57], there are diverse environmentally safe strains related to *B. cereus* that produce metabolites with antimicrobial properties and plant-stimulating traits [58]. For example, the *B. cereus* YN917 strain was isolated from a healthy rice leaf and has significant antifungal activity against *Magnaporthe oryzae*, as well as plant growth-promoting activity [59]. Another bacterial isolate is the *B. cereus* CF4-51 strain, which was isolated from sunflower rhizosphere. Its inhibitory ability against the plant pathogen *Sclerotinia sclerotiorum* was demonstrated to be mediated by VOCs and lipopeptides [60]. Also, as an endophyte of Bhut Jolokia (*Capsicum chinense* Jacq.), *B. cereus* has been isolated, and its characterization showed inhibition of mycelial growth of *C. gloeosporioides* [55]. Other relevant mechanisms against phytopathogenic fungal strains have been described in *B. cereus*, for example, ribosomally as well as non-ribosomally synthesized antimicrobial peptides (NRPs) such as bacillomycin, bacilysin, surfactin, iturin, fengycin, plipastatin and many others have strong activity against *A. solani*, *Leptosphaeria maculans*, *S. sclerotiorum*, *F. solani*, *F. oxysporum*, *C. gloeosporioides*, *Cercospora lactucae-sativae*, *M. oryzae*, *R. solani*, and *S. rolfsii* [58]. Diverse strains of *B. cereus* promote plant growth and can trigger ISR responses against pathogenic fungi mediated by the salicylic acid, jasmonic acid, and ethylene pathways [58]. Therefore, based on these findings, this may be the first report that the bacterial strain characterized in this study and identified as *B. tropicus* CH13 through genome analysis may be associated with the antifungal properties of chili seeds. According to the results of the phylogenetic and ANI analysis, *B. tropicus* CH13 showed high ANI values (>95%) with the strains *B. tropicus* N24 and *B. cereus* Q1. The first strain was isolated from sediment in the South China Sea [56], and the second strain was isolated from a deep-subsurface oil reservoir in the Daqing oil field in northeastern China. They have the ability to produce biosurfactants and survive in extreme environments [61]. Other *B. tropicus* strains have been isolated from soil. For example, *B. tropicus* EMB20 produces an interesting β-lactamase with high activity [62]. Related to this, genome analysis detected the β-lactamase gene in *B. tropicus* CH13, but the phenotypic results for β-lactam resistance revealed an unfunctional gene. In this sense, additional genetic analysis is necessary to better understand this unfunctional gene. Also, *B. tropicus* Gxun-17 is an interesting strain that was isolated from sludge at a marine duck farm in the Beibu Gulf in Guangxi, China. It produces a keratinase enzyme involved in feather degradation [63]. In addition, some strains of *B. tropicus* have been studied related to the biodegradation of xenobiotic compounds such as pentachlorophenol [64], glyphosate [65], and low-density polyethylene [66]. Interestingly, there is an important report on the *B. tropicus* JMT strain, which was isolated from Chinese soft-shelled turtles (*Tryonyx sinensis*) and causes an anthrax-like disease [67]. Therefore, these findings revealed that many metabolic features of *B. tropicus* CH13 remain unexplored, and more efforts are necessary to understand the production of toxins associated with the *nheA*, *nheB*, and *nheC* genes (non-hemolytic enterotoxins); the *hblA*, *hblC*, and *hblD* genes (hemolytic entorotoxins); and cytotoxin K (*cytK*) to evaluate potential environmental risk as well as genomic comparisons with other antibiotic-resistant strains and to understand the antibiotic sensibility shown in this study and the evolution of this bacterial strain.

Chili´s seed-associated bacteria represent a significant motivation to explore the vital impacts of on plant–bacteria interactions and their ecological roles during colonization competition against phytopathogens that cause diseases in chili cultivars. Recent efforts in different countries have been focused on biocontrol as an alternative to using chemical fungicides on chili cultivars to avoid toxicity in the environment [68,69]. For example, the endophyte *B. velezensis* LY7, which was isolated from pepper leaves, possessed the ability to control anthracnose disease caused by *C. scovillei* in pepper plants by stimulating hormone synthesis to enhance disease resistance and promote plant growth in greenhouse and in vitro assays [70]. Another study demonstrated that the *B. subtilis* AKP strain exhibited antagonistic activity against the chili anthracnose fungus *C. capsici* via the production of homologues of surfactin, iturin, and fengycin lipopeptide biosurfactant as well as plant growth promotion in chili plants in both in vitro assays and in vivo pot experiments [71]. Biocontrol of *F. languescens* CE2 isolated from a Jalapeño chili (*Capsicum annuum* var. Jalapeño) with wilt symptoms was confronted with *B. cabrialesii* subsp. *tritici* TSO2^T^, and the reduction in mycelial growth was severely affected by this bacterial strain, probably due to associated genes related to bacillaene, subtilosin A, fengycin, surfactin, bacillibactin, bacilysin, and sporulation killing factor [72]. Also, experiments in pots and fields carried out with *B. subtilis* PTS-394 demonstrated excellent biocontrol of *F. solani*, which causes pepper root rot, through lipopeptide production as well as the ability to promote pepper seed germination and plant height [73]. These findings show that diverse species from the *Bacillus* genus and their alternative metabolic pathways produce diverse metabolites that can be used for the biocontrol of phytopathogens. Therefore, in this study, biocontrol using chili seed-surface-associated bacteria was assayed principally through VOC production to find potential strains with future applications in sustainable agriculture. Additional work is currently in progress using both bacterial strains identified in this study to evaluate their effects on plant responses as well as additional metabolites and lytic enzymes with biocontrol activity against phytopathogens (in preparation). However, as a first step, characterizing the VOC production in these bacterial strains was important for understanding their ecological roles. The results showed that common classes of VOCs (alcohols, aldehydes, acids, pyrazines, ketones, hydrocarbons, nitro compounds, thiols, and other compounds) detected in the *Bacillaceae* family [48] were produced in both strains, but interestingly some common VOCs such as 2,5-dimethyl pyrazine, acetoin, nonanoic acid, n-decanoic acid, octanoic acid, methyl pyrazine, 2,3-butanedione, piperonal, and 1-butanol were produced with high relative abundance values in both strains at the evaluated timepoint (Figure 7 and Figure 8). However, differences in their VOC compositions and relative abundance percentages led to differences in their mycelial growth effects in *A. alternata. B. altitudinis* CH05 showed 65% inhibition, while *B. tropicus* CH13 showed 28.88% inhibition. Although individual VOCs were found to have significant effects against phytopathogenic microorganisms [48], complex mixtures of VOCs produced with different concentrations can also have major impacts, as each specific compound can affect a cell via multiple routes [48]. For example, in this study (Figure 9 and Figure 10), a mixture of 2,5-dimethyl pyrazine, acetoin, and benzaldehyde had a large effect on the mycelial growth of the tested fungal strains compared with acetoin and 2,5-dimethyl pyrazine when they were applied individually at higher volume concentrations, while that 2-butanone was less effective against *S. rolfsii* in all volume concentrations compared with *R. solani*. In the case of treatment 13 (T13) against *S. rolfsii*, 2,5-dimethyl pyrazine was in mayor volume concentration compared with the treatments T12 and T14 and demonstrated a major growth inhibition. On the other hand, 2,5-dimethyl pyrazine showed high mycelial growth inhibition against *R. solani* in the treatment T1 with lower volume concentration compared with *S. rolfsii*, and probably due to this difference, the treatments T12, T13 and T14 against *S. rolfsii* showed complete mycelial growth inhibition. These results demonstrate that 2,5-dimethyl pyrazine and acetoin, which were present at high relative abundance in the fermentation cultures and with mycelial growth inhibition effects, are important VOCs produced by both bacterial strains. In addition, suitable complex mixtures of VOCs can be used at lower concentrations to reduce their ecological impacts when high concentrations of individual VOCs used to control microorganisms have toxic effects on plants and animals [74]. In this study, it was particularly important that *B. tropicus* CH13 produced acetoin with a high relative abundance of 32.77%. In accordance with increasing interest in biological production, it could be a valuable bacterial strain used to produce this compound, with applications in agriculture, food, medicine, and the pharmaceutical industry [75,76].

Effective colonization routes of plant-seeds by microorganisms during early stages of plant development is an important evolutive mechanism with significant ecological roles. Thus, for example seed microbiome will ensure the permanence in the next plant generation as part of life cycle and regulate seed germination, plant development, stress tolerance or colonization competence with other microorganisms to regulate disease protection [77,78]. However, environmental conditions for vertical and horizontal transmission of seed-associated microorganism also make necessary an evaluation and characterization of potential risk associated with metabolic traits of these microorganisms, such as virulence genes or antibiotic resistance mechanisms, which can be spread if these microorganisms are used as a biocontrol alternative [79]. In our study, both bacterial strains were evaluated for antibiotic resistance, where *B. altitudinis* CH05 showed resistance to five antibiotics, while that *B. tropicus* CH13 was sensitive to all antibiotics, but in the genome of this last bacterial strain has virulence genes related with toxins production. Although *B. tropicus* CH13 can be potentially risky when used as biocontrol, its metabolite compounds can be produced as a safe alternative.

## 5. Conclusions

We characterized two bacterial strains isolated from chili seeds: *B. altitudinis* CH05 and *B. tropicus* CH13. Both strains showed strong mycelial growth inhibition against *Sclerotinia* sp., *R. solani*, and *S. rolfsii*, but there were some differences when assayed with *A. alternata* through the production of VOCs. Their VOC profiles revealed some common compounds identified in the *Bacillaceae* family with proven antifungal activity. In the case of *B. tropicus* CH13, to the best of our knowledge, this is the first report on this bacterial strain associated with the biocontrol activity and isolated from chili seeds through the production of VOCs. In particular, acetoin showed a high relative abundance percentage in *B. tropicus* CH13, and this could be a suitable bacterial strain for acetoin production. Further exploration of ecological roles and plant interactions could provide better alternatives for the biocontrol of phytopathogens in sustainable agriculture using these bacterial strains characterized as well as the rest of bacterial strains isolated.

## Figures and Tables

**Table 1:** Seed-surface-associated bacteria obtained from *Capsicum* spp.

Chili Cultivar	Specie	Region	Number of Bacterial Isolates
Serrano	*Capsicum annuum* L.	Querétaro	9
Jalapeño	*Capsicum annuum* L.	Querétaro	2
Pimiento	*Capsicum annuum* L.	Querétaro	2
Poblano	*Capsicum annuum* L.	Querétaro	3
Habanero	*Capsicum chinense*	Querétaro	5
Poblano	*Capsicum annuum* L.	Michoacán	5
Jalapeño	*Capsicum annuum* L.	Michoacán	7
Manzano	*Capsicum pubescens*	Michoacán	8
Píquin	*Capsicum annuum* L.	Michoacán	3
Serrano	*Capsicum annuum* L.	Michoacán	6
Habanero	*Capsicum chinense*	Michoacán	3
Húngaro	*Capsicum annuum* L.	Michoacán	9
Chilaca	*Capsicum annuum* L.	Michoacán	8
Habanero	*Capsicum chinense*	Yucatán	2
Húngaro	*Capsicum annuum* L.	Yucatán	1
Dulce	*Capsicum annuum* L.	Yucatán	3

**Table 2:** Genome analysis of *B. altitudinis* CH05 and *B. tropicus* CH13.

Characteristics	Source	*B. altitudinis* CH05	*B. tropicus* CH13
Genome length (bp)	PATRIC	3,687,876 bp	5,283,743 bp
Contigs	PATRIC	18	54
Proteins with functional assignments	PATRIC	3050	4265
Hypothetical proteins	PATRIC	844	1243
rRNA	PATRIC	3	4
tRNA	PATRIC	68	73
Proteins with pathway assignments	KEGG	683	830
GC content	PATRIC	41.25%	35.24%
N50 length (bp)	PATRIC	973,713	381,948
Virulence factors	Victors	2	12
Virulence factors	VFDB	0	9
Antibiotic resistance	PATRIC	40	48
Antibiotic resistance	CARD	2	7
Antibiotic resistance	NDARO	2	5
Transporters	TCDB	65	52

## Data Availability

The data presented in this work are available from the corresponding authors upon request.

## Supplementary Materials

The following supporting information can be downloaded at https://www.mdpi.com/article/10.3390/microorganisms12101943/s1, Table S1. Pairwise comparison of Average Nucleotide Identities (ANIs) of *B. altitudinis* CH05 species. ANI values ≥ 95% are shown in red. Table S2. Pairwise comparison of Average Nucleotide Identities (ANIs) of *B. tropicus* CH13 and CH05. ANI values ≥ 95% are shown in red. Table S3. Identification of 64 VOCs produced by *B. altitudinis* CH05 and detected via HS-SPME-GC-MC. Table S4. Identification of 53 VOCs produced by *B. tropicus* CH13 and detected via HS-SPME-GC-MC. Figure S1. Antibiotic resistance genes of *B. altitudinis* CH05. The AMR mechanism and its related genes are indicated by the corresponding color. Figure S2. Antibiotic resistance genes of *B. tropicus* CH13. The AMR mechanism and its related genes are indicated by the corresponding color.
