# Supplementary material for: Biocontrol Activity of Bacillus altitudinis CH05 and Bacillus tropicus CH13 Isolated from Capsicum annuum L. Seeds against Fungal Strains
Source: Microorganisms. 2024 Sep 25;12(10):1943. doi: 10.3390/microorganisms12101943 (PMC11509363; doi:10.3390/microorganisms12101943)
Supplement: Supplementary file 1 [file microorganisms-12-01943-s001.zip › microorganisms-3201611-supplementary.pdf]

Supplementary Results

ANI VALUES > 95%	*B. altitudinis* CH05	*B. altitudinis* DSM 26896	*Bacillus altitudinis RIT380*	*Bacillus altitudinis* NIO-1130 [T]	*Bacillus altitudinis* B4133	*Bacillus altitudinis* W3	*Bacillus altitudinis* 41KF2b [T]	*Bacillus safensis* B4129	*Bacillus safensis* CFA06	*Bacillus safensis* RIT372	*Bacillus pumilus* B4127	*Bacillus pumilus* CB01	*Bacillus atrophaeus* NRS 1221A	*Bacillus atrophaeus* C89	*Bacillus subtilis* BS34A	*Bacillus subtilis* QB928	*Bacillus velezensis* FZB42	*Bacillus velezensis* M27	*Bacillus sonorensis* L12	*Bacillus sonorensis* NBRC 101234 = KCTC 13918 NBRC 101234 [T]
B. altitudinis CH05		97.87	97.85	98.21	97.99	98.36	98.22	88.54	88.57	88.57	88.49	88.48	69.99	69.93	70.01	70.01	69.51	69.52	69.46	69.4
B. altitudinis DSM 26896	97.86		98.25	97.86	97.98	97.83	97.88	88.61	88.65	88.66	88.39	88.43	69.96	69.9	69.98	70.00	69.51	69.50	69.45	69.46
Bacillus altitudinis RIT380	97.68	98.03		97.66	97.87	97.64	97.66	88.48	88.52	88.57	88.42	88.41	70.10	70.02	70.02	70.01	69.8	69.64	69.48	69.6
Bacillus altitudinis NIO-1130 [T	98.24	97.94	97.89		98.08	98.30	98.57	88.55	88.58	88.61	88.38	88.37	70.12	70.03	70.07	70.08	69.49	69.54	69.45	69.42
Bacillus altitudinis B4133	98.09	98.01	98.12	98.15		98.12	98.11	88.62	88.68	88.63	88.46	88.43	69.99	69.9	69.96	69.97	69.54	69.5	69.36	69.33
Bacillus altitudinis W3	98.22	97.73	97.78	98.15	97.91		98.19	88.58	88.54	88.52	88.48	88.45	70.27	69.9	70.30	70.31	69.82	69.8	69.78	69.73
Bacillus altitudinis 41KF2b [T]	98.3	97.95	97.83	98.58	98.07	98.35		88.55	88.65	88.62	88.36	88.43	70.08	70.00	70.07	70.06	69.65	69.63	69.58	69.51
Bacillus safensis B4129	88.48	88.51	88.55	88.54	88.49	88.6	88.51		96.00	96.06	91.18	91.21	70.07	69.99	70.08	70.08	69.70	69.66	69.46	69.43
Bacillus safensis CFA06	88.56	88.64	88.6	88.62	88.62	88.56	88.64	96.08		98.34	91.24	91.24	69.92	69.88	69.96	69.96	69.46	69.42	69.47	69.45
Bacillus safensis RIT372	88.6	88.64	88.62	88.61	88.6	88.58	88.63	96.09	98.42		91.29	91.28	70.24	70.11	70.15	70.12	69.86	69.67	69.59	69.56
Bacillus pumilus B4127	88.39	88.33	88.39	88.34	88.36	88.47	88.33	91.11	91.15	91.19		98.4	69.97	69.87	69.94	69.93	69.57	69.48	69.46	69.46
Bacillus pumilus CB01	88.47	88.38	88.52	88.4	88.45	88.46	88.45	91.17	91.23	91.23	98.45		70.22	70.08	70.01	70.03	69.83	69.68	69.48	69.6
Bacillus atrophaeus NRS 1221A	70.41	70.28	70.35	70.23	70.34	70.35	70.38	70.27	70.24	70.44	70.25	70.21		99.96	79.21	79.18	76.82	76.78	72.43	75.45
Bacillus atrophaeus C89	69.9	69.82	69.89	69.91	69.89	69.87	69.89	69.97	69.83	69.94	69.85	69.82	99.97		79.06	79.00	76.64	76.68	72.22	72.24
Bacillus subtilis BS34A	70.32	70.27	70.39	70.18	70.25	70.39	70.36	70.34	70.43	70.46	70.29	70.27	79.25	78.98		99.84	76.44	76.31	72.66	72.69
Bacillus subtilis QB928	70.39	70.30	70.35	70.24	70.33	70.41	70.39	70.28	70.38	70.35	70.29	70.27	79.29	79.02	99.96		76.40	76.29	72.67	72.70
Bacillus velezensis FZB42	70.08	69.93	70.11	69.85	70.04	70.00	70.03	69.91	70.07	70.17	70.09	69.96	76.81	76.47	76.35	76.36		97.61	72.14	72.18
Bacillus velezensis M27	69.62	69.52	69.57	69.52	69.58	69.6	69.59	69.50	69.49	69.54	69.50	69.45	76.64	76.53	76.11	76.10	97.62		71.72	71.72
Bacillus sonorensis L12	69.29	69.19	69.14	69.29	69.19	69.32	69.24	69.19	69.17	69.30	69.27	69.25	72.01	71.93	72.01	72.02	71.44	71.41		99.56
Bacillus sonorensis NBRC 101234 = KCTC 13918 NBRC 101234 [T]	69.14	69.15	69.21	69.21	69.17	69.2	69.16	69.26	69.25	69.32	69.21	69.15	72.14	72.07	72.08	72.08	71.58	71.51	99.63	

Table S1. Pairwise comparison of Average Nucleotide Identity (ANI) for *B. altitudinis* CH05 species. ANI values ≥95% are shown in red.

ANI VALUES > 95%	*B. tropicus* CH13	*B. tropicus* N24[T]	*B. anthracis* str. A0174	*B. anthracis* str. H9401	*B. anthracis* HYU01	*B. anthracis* BA1015	Bacillus cereus TIAC219	*Bacillus cereus* D17	*Bacillus cereus AH820*	*Bacillus cereus* Q1	*Bacillus thuringiensis* T01-328	*Bacillus thuringiensis* YBT-1518	*Bacillus thuringiensis* YC-10	*Bacillus thuringiensis* HD-789
B. tropicus CH13	*	95.67	94.30	94.39	94.4	94.39	91.2	94.13	94.4	95.81	91.33	91.27	91.30	91.22
B. tropicus N24 [T]	95.69	*	94.32	94.39	94.37	94.39	91.09	94.09	94.36	94.92	91.29	91.29	91.29	91.12
B. anthracis str. A0174	94.44	94.36	*	99.97	99.93	99.97	90.78	96.96	98.25	94.61	90.93	90.95	90.76	90.75
B. anthracis str. H9401	94.45	94.24	99.86	*	99.91	99.97	90.76	96.89	98.16	94.53	90.89	90.89	90.74	90.76
B. anthracis HYU01	94.49	94.32	99.82	99.92	*	99.93	90.84	96.93	98.24	94.63	90.96	91.02	90.85	90.88
B. anthracis BA1015	94.46	94.31	99.85	99.96	99.92	*	90.89	96.92	98.22	94.65	90.96	90.96	90.78	90.84
Bacillus cereus TIAC219	90.72	90.53	90.05	90.13	90.14	90.13	*	90.11	90.32	90.26	95.34	94.97	94.82	97.88
Bacillus cereus D17	94.25	94.15	96.82	96.87	96.91	96.90	90.8	*	97.18	94.55	90.72	90.78	90.79	90.73
Bacillus cereus AH820	94.36	94.30	98.07	98.13	98.13	98.14	90.87	97.03	*	94.56	90.90	91.00	90.83	90.86
Bacillus cereus Q1	95.76	94.81	94.45	94.52	94.50	94.52	90.89	94.44	94.61	*	90.99	91.02	90.97	90.90
Bacillus thuringiensis T01-328	90.72	90.73	90.20	90.27	90.29	90.29	95.51	89.97	90.27	90.30	*	97.67	95.58	95.31
Bacillus thuringiensis YBT-1518	90.80	90.75	90.29	90.36	90.36	90.36	94.79	90.02	90.38	90.45	97.18	*	94.61	94.94
Bacillus thuringiensis YC-10	90.91	90.83	90.09	90.14	90.16	90.17	94.77	90.07	90.05	90.20	95.69	94.64	*	94.35
Bacillus thuringiensis HD-789	91.09	90.88	90.50	90.58	90.58	90.60	98.46	90.48	90.63	90.75	95.66	95.64	94.94	*

Table S2. Pairwise comparison of Average Nucleotide Identity (ANI) for *B. tropicus* CH13 CH05. ANI values ≥95% are shown in red.

Compounds	Retention Time (min)	Relative Peak Area (%)	Chemical Classes	Compounds	Retention Time (min)	Relative Peak Area (%)	Chemical Classes
1,2-Bis(difluoroamino) ethane	0.246	1.03	Hydrocarbon	Trimethyl-	34.372	1.22	Pyrazine
Carbon dioxide	4.053	2.13	Hydrocarbon	Nonanal	34.857	0.65	Aldehyde
n-Hexane	4.404	2.29	Hydrocarbon	Acetic acid	36.043	2.02	Acid
Methanethiol	4.648	0.73	Thiols	3-Ethyl-2,5-dimethyl pyrazine	36.539	2.68	Pyrazine
Acetaldehyde	4.782	1.07	Aldehyde	4-Heptanol, 2,6-dimethyl-	37.686	1.1	Alcohol
Heptane	4.903	2.78	Hydrocarbon	1-Hexanol, 2-ethyl-	38.77	1.15	Alcohol
Acetone	5.988	1.04	Ketone	Oxalic acid, 2TMS derivative	38.993	0.27	Acid
2-Chloro-4,8-dimethylquinoline	7.144	0.46	Other compounds	Benzaldehyde	39.474	1.99	Aldehyde
Butanal	7.301	0.42	Aldehyde	Pyrazine, 2-methyl-3-(2-propenyl)-	40.468	0.63	Pyrazine
2-Butanone	7.963	0.61	Ketone	1-Octanol	41.608	1.62	Alcohol

Butanal, 2-methyl-	8.526	0.66	Aldehyde	Pentanoic acid	43.864	0.44	Acid
Butanal, 3-methyl-	8.689	1.67	Aldehyde	Benzeneacetaldehyde	44.557	0.93	Aldehyde
Ethanol	9.009	1.36	Alcohol	2-Furanmethanol	44.834	0.92	Alcohol
2,3-Butanedione	11.55	2.24	Ketone	Butanoic acid, 3-methyl-	45.78	3.54	Acid
Trichloroethylene	12.247	0.36	Other compounds	Pyrazine, 2,5-dimethyl-3-(3-methyl butyl)-	46.143	0.69	Pyrazine
Naphtho [3,4:2,3] bornene	13.73	0.42	Other compounds	1-Propanol, 3-(methylthio)-	47.586	0.17	Alcohol
Methyl thiolacetate	15.049	0.27	Thiols	Pyrazine, 2-methoxy-3-(1-methylpropyl)-	50.85	0.58	Pyrazine
Disulfide dimethyl	16.144	1.03	Other compounds	Nonyl chloroformate	51.863	0.61	Other compounds
1-Butanol	21.126	1.34	Alcohol	1-Oxa-4-thiaspiro [4.4]nonane	56.958	1.15	Ketone
2-Hydroxyphenethyl alcohol, 2TMS derivative	23.203	0.39	Alcohol	Benzyl alcohol	58.039	0.27	Alcohol
7-Oxabicyclo [2.2.1]heptane, 1-methyl-4-(1-methylethyl)-	23.464	0.68	Hydrocarbon	Phenylethyl Alcohol	61.505	0.55	Alcohol
2-Ethylcyclo pentanone	23.817	0.36	Ketone	α-D-Galactopyranoside, methyl	67.569	0.34	Hydrocarbon
1,3-Diazine	24.149	2.4	Other compounds	Benzaldehyde, 4-methoxy-	70.827	0.3	Aldehyde
3-Methyl-1-butanol	24.627	4.43	Alcohol	Octanoic acid	74.863	2.46	Acid
Methylpyrazine	27.183	2.24	Pyrazine	Nonanoic acid	80.275	6.72	Acid
Acetoin	28.404	11.9	Ketone	Piperonal	81.377	2.14	Aldehyde
2-Propanone, 1-hydroxy-	28.926	0.2	Ketone	8-Methylnonanoic acid	83.025	0.45	Acid
2,5-dimethylpyrazine	30.247	13.31	Pyrazine	n-Decanoic acid	84.841	2.67	Acid
Oxalic acid, 2TMS derivative	31.743	0.28	Acid	2,4-Di-tert-butyl phenol	86.147	0.5	Alcohol
1-Hexanol	32.229	1.17	Alcohol	D-Mannopyranose	92.523	0.26	Hydrocarbon
Dimethyl trisulfide	33.047	0.39	Other compounds	Benzaldehyde, 3-hydroxy-4-methoxy-	93.227	0.3	Aldehyde
2-Ethyl-5-methyl pyrazine	33.846	0.82	Pyrazine	Phthalic acid, hex-3-yl isobutyl ester	94.24	0.24	Ester

Tabla S3. Identification of 64 VOC´s produced by the *B. altitudinis* CH05 and detected by HS-SPME-GC-MC.

Compounds	Retention Time (min)	Relative Peak Area (%)	Chemical Classes	Compounds	Retention Time (min)	Relative Peak Area (%)	Chemical Classes
**Carbon dioxide**	4.048	1.1	Hydrocarbon	Pyrazine, 2-ethyl-5-methyl-	33.839	1.19	Pyrazine
**Pentane**	4.188	0.86	Other compound	Pyrazine, trimethyl-	34.372	0.98	Pyrazine
**n-Hexane**	4.405	1.51	Other compound	Nonanal	34.861	0.71	Aldehyde
**Methanethiol**	4.649	0.81	Thiol	Acetic acid	36.155	0.18	Acid
**Heptane**	4.907	2.22	Hydrocarbon	Pyrazine, 3-ethyl-2,5-dimethyl-	36.502	0.67	Pyrazine
**Acetone**	5.989	0.68	Ketone	1-Hexanol, 2-ethyl-	38.749	0.51	Alcohol
**Silane, dimethyl (dimethyl(2-decylxy) silyloxy)propoxy- 6-Nitrocoumarin**	7.146	0.9	Other compound	Oxalic acid, 2TMS derivative	39.003	0.22	Acid
**2-Butanone**	7.976	0.48	Ketone	Benzeneacetonitrile, .α-phenyl-	39.678	0.62	Nitro compound
**2,3-Butanedione**	11.577	10.38	Ketone	Pyrazine, 2-methyl-6-(1-propenyl)-	40.474	0.37	Pyrazine
**Trichloroethylene**	12.274	0.89	Hydrocarbon	2-Acetylthiazole	44.591	0.29	Thiol
**2-Mercapto-4-phenylthiazole**	13.771	0.45	Thiol	Acetophenone	44.855	0.21	Ketone
**Butanoic acid, ethyl ester**	15.028	0.33	Ester	Serine	45.856	0.74	Other compound
**Disulfide, dimethyl**	16.169	0.22	Other compound	2,3-Dimethyl-5-isopentylpyrazine	46.151	0.34	Pyrazine
**1-Butanol, 3-methyl-, acetate**	20.175	0.2	Alcohol	D-Serine.β.-D-Ribopyranoside, methyl	57.003	0.7	Other compound
**1-Butanol**	21.065	1.3	Alcohol	Phenylethyl Alcohol	61.529	1.06	Alcohol
**Disilathiane, hexamethyl-**	23.248	0.22	Other compound	.β-l-Arabinopyranoside, methyl	67.565	0.44	Other compound
**7-Oxabicyclo[2.2.1] heptane, 1-methyl-4-(1-methylethyl)-**	23.468	0.24	Hydrocarbon	Octanoic acid	74.863	2.35	Acid
**1,3-Diazine**	24.146	1.56	Nitro compound	2-Nonadecanone	77.452	0.43	Ketone
**1-Pentanol**	24.606	3.78	Alcohol	11-Dodecen-2-one, 2-Nonadecanone	78.205	0.28	Ketone
**Pyrazine, methyl-**	27.176	1.82	Pyrazine	Nonanoic acid	80.269	6.53	Acid
**Acetoin**	28.436	32.77	Ketone	Piperonal	81.38	1.17	Aldehyde
**Acetoin**	28.91	0.69	Ketone	Methyl anthranilate	81.916	0.15	Acid
**Pyrazine, 2,5-dimethyl-**	30.248	12.33	Pyrazine	n-Decanoic acid	84.867	1.67	Acid

**Pyrazine, 2,5-dimethyl-, 1-Cyclobutanone, 2-(2-methyl-1-propenyl)**	30.871	0.4	Pyrazine	Pyrido[3,4-d] pyrimidine-2,4(1H,3H)-dione, 6,8-dimethyl-	86.145	0.34	Nitro compound
**Malonic acid, bis(2-trimethylsilylethyl ester**	31.756	0.31	Ester	Pyridine, 2-ethyl-6-methyl-	87.601	0.23	Nitro compound
**Heptanoic acid, ethyl ester**	32.142	0.68	Ester	4-Trifluoromethyl benzoic acid.exadecyl ester	93.684	0.24	Ester
				1,2-Benzene dicarboxylic acid, bis(2-methylpropyl) ester	94.242	0.27	Ester

Table S4. Identification of 53 VOC´s produced by *B. tropicus* CH13 and detected by HS-SPME-GC-MC.

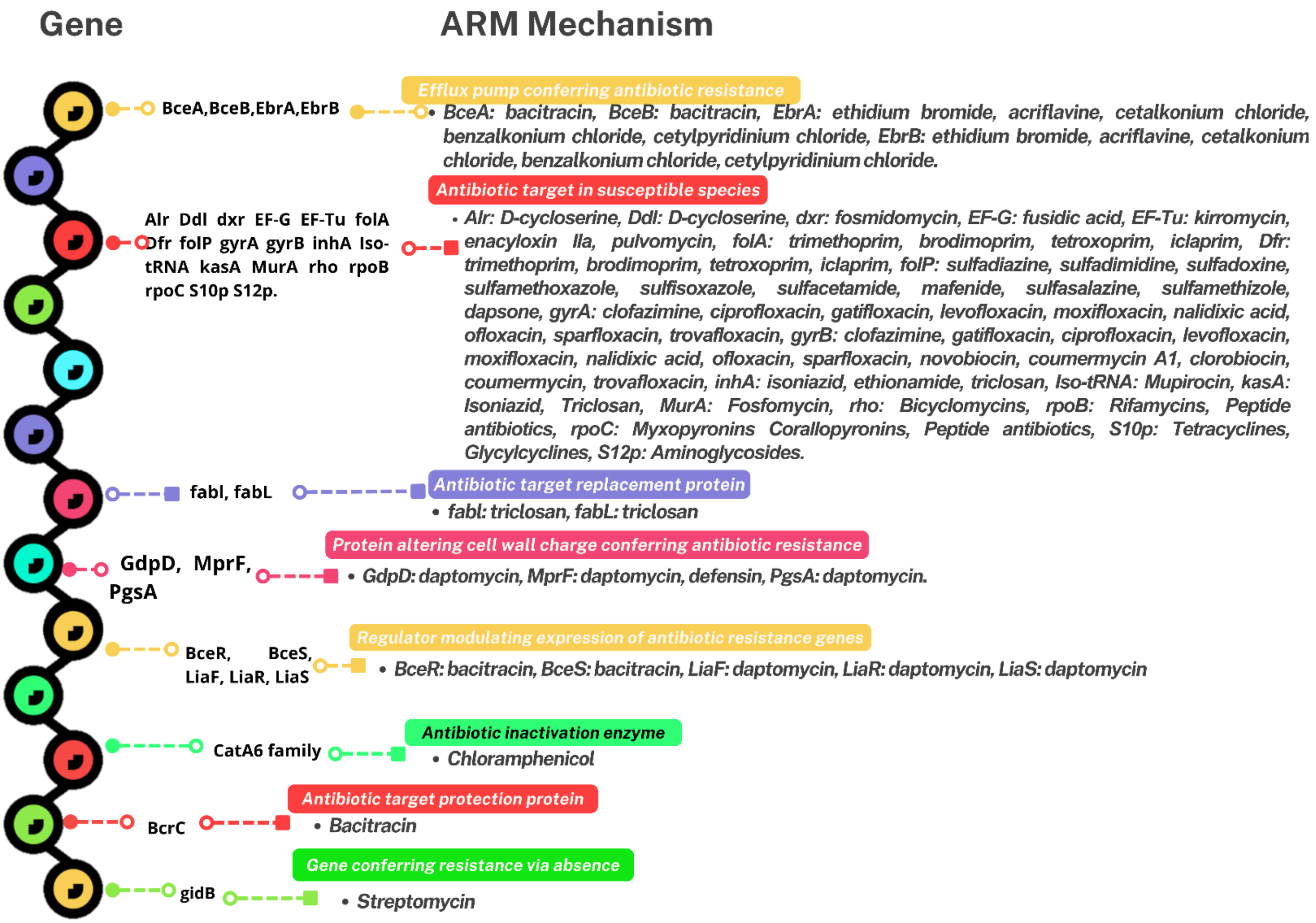

Figure S1. Antibiotic resistance genes for *B. altitudinis* CH05. The AMR mechanism and its related genes are indicated by the corresponding color.

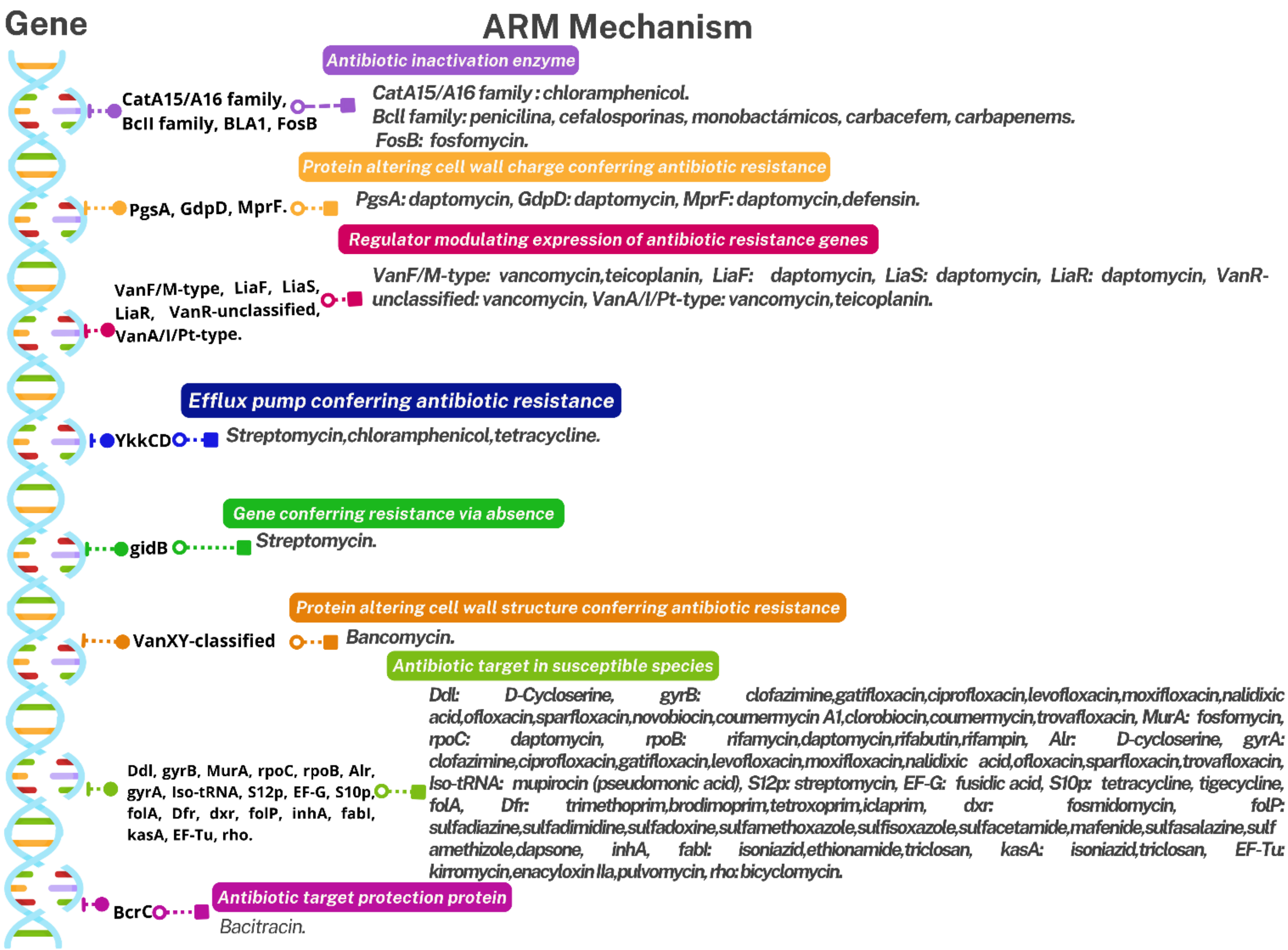

Figure S2. Antibiotic resistance genes for *B. tropicus* CH13. The AMR mechanism and its related genes are indicated by the corresponding color.
